# 

**Published:** 1905-03-04

**Authors:** 


### NOTICE TO CORRESPONDENTS.

All MSS., Letters, Books for Review, and other matters intended for the Editor should be addressed The Editor, "The Hospital." The Hospital Buildings, 28 & 29 Southampton Street, Strand, W.C.

The Editor cannot undertake to return rejected MS., even when accompanied by stamped directed envelope.

**Notice to Advertisers and Subscribers.**

All Advertisements, Orders for Copies of The Hospital, and Business Communications should be addressed to **THE MANAGER (Not to the Editor),** The Hospital, 28 & 29 Southampton Street, Strand, London, W.C.

The Cost of Subscription, post free, is as follows:—Per week, 3½d.; per quarter, 3s. 3d.; per half-year, 6s. 6d.; per annum, 13s. Foreign and Colonial:—Per annum, 19s. 6d., payable, in all cases, in advance.

**NOTICE.—Vol. XXXVI. of "The Hospital" (April to September 1904), in handsome cloth binding, is now on sale at the office of this Journal, price 10s. 6d. Cases for binding the Numbers contained in this Volume 2s. Index to Vol. XXXVI., 3½d., post free.**

**The Monthly part for March is now ready. Price 1s., by post, 1s. 3d.**

### BOOKS RECEIVED.

G. Street and Co., Limited.
"Street's Newspaper Directory, 1905."

Adlard and Son.
"The British Journal of Children's Diseases." Vol. I.

David Nutt.
"To Windward." By Henry C. Rowland.

### MEDICAL APPOINTMENTS.

**Walter Chapman**, M.B., B.Ch.Birm., L.R.C.P.Lond., M.R.C.S., has been appointed Public Vaccinator for the Fourth District of the Totnes (Devon) Union. **Herbert G. G. Cook**, M.D.Lond., F.R.C.S.Eng., D.P.H.Camb., has been appointed Medical Officer to H.M. Prison at Cardiff. **Douglas Douglas-Crawford**, M.B.Edin., F.R.C.S.Eng., has been appointed Honorary Consulting Surgeon to the Liverpool Dental Hospital. **H. W. Evans**, M.R.C.S.Eng., L.R.C.P.Edin., has been appointed Certifying Surgeon under the Factory and Workshop Act for the Littleport District of the county of Cambridge. **G. C. B. Hawes**, M.R.C.S., L.R.C.P.Lond., has been appointed Certifying Surgeon under the Factory and Workshop Act for the Pangbourne and Whitchurch District of the counties of Berks and Oxford. **Percy Wheeler Kent**, M.R.C.S., L.R.C.P., D.P.H.Camb., has been appointed Surgeon to the Glamorgan County Police at Barry. **Francis Henry Knaggs**, M.R.C.S., L.R.C.P.Lond., has been appointed Ophthalmic and Aural Surgeon to the Huddersfield Infirmary. **F. A. Mills**, M.B., B.C.Cantab., has been appointed Assistant Anæsthetist to the Royal National Orthopædic Hospital. **H. B. Milsome**, M.D.St. And., has been appointed Certifying Surgeon under the Factory and Workshop Act for the Chertsey District of the county of Surrey. **R. W. C. Pierce** M.D., B.Sc.Lond., D.P.H.Camb., has been appointed Medical Officer of Health of the Borough of Guildford. **George Stevens Pope**, L.R.C.P. & S.Edin., L.F.P.S.Glasg., has been appointed Medical Superintendent at the Somerset and Bath Asylum, Wells.

**"The Hospital" Institutional Library.**

**"Hospitals and Asylums of the World." 4 Vols., with a Portfolio of Plans, complete £12 12s.**

**"Burdett's Hospitals and Charities, 1904." 5s. net.**

**"Burdett's Official Nursing Directory." 8s. net.**

**"Cottage Hospitals, General, Fever, and Convalescent." 10s. 6d.**

**"Uniform System of Accounts for Hospitals and Public Institutions." New Edition. 4s. net.**

**"Hospital Expenditure: The Commissariat." 2s. 6d.**

**"A Legal Handbook for the Use of Hospital Authorities." 1s. 6d.**

**"The Cottage Hospital Case Book or Register of Patients." 10s. and 12s. 6d.**

**All these are published by the Scientific Press, Ltd., and may be obtained through any bookseller, or direct from the publishers, 28 and 29 Southampton Street, Strand, London, W.C.**

### NEW BOOKS AND SMALL MANUALS for NURSES

**AN ELEMENTARY TREATISE ON THE LIGHT TREATMENT FOR NURSES.** 2/6 net, 2/9 post free.
By J. H. SEQUEIRA, M.D., Physician in charge of the Skin Department of the London Hospital.

**A COMPLETE HANDBOOK OF MIDWIFERY.** 6/- net, 6/4 post free.

Profusely Illustrated.

By J. K. WATSON, M.D.

**THE MODERN NURSING OF CONSUMPTION.** 1/- net, 1/2 post free.

By Dr. JANE WALKER.

**HOW TO BECOME A CERTIFIED MIDWIFE.** 1/- net, 1/1 post free.

By E. APPEL, M.B.

### THE BURDETT SERIES.

**Post free, 1s. each. Or the Eleven Volumes complete, post free, 10s.**

No. 1.—**PRACTICAL HINTS ON DISTRICT NURSING.**
By Miss Amy Hughes.

No. 2.—**HOSPITAL AND PRIVATE NURSING.**
By Miss S. E. Orme.

No. 3.—**THE MIDWIVES' POCKET BOOK** (with Prefatory Chapter on the Midwives Bill).
By Miss Honnor Morten, Author of "The Nurses' Dictionary," &c., &c.

No. 4.—**FEVERS AND INFECTIOUS DISEASES:** their Nursing and Practical Management.
By William Harding, M.D. Ed., M.R.C.P. Lond., Author of "Mental Nursing," &c.

No. 5.—**NOTES ON PHARMACY AND DISPENSING FOR NURSES.** New and Revised Edition.
By C. J. S. Thompson.

No. 8.—**HOME NURSING OF SICK CHILDREN.**
By J. D. E. Mortimer, M.B., F.R.C S.

No. 9.—**MENTAL NURSING.**
By William Harding, M.B. Ed., M.R.C.P. Lond.

No. 10.—**SICK NURSING AT HOME.**

By Miss L. G. Moberly.

No. 11.—**GYNÆCOLOGICAL NURSING.**

By G. A. Hawkins-Ambler, F.R.C.S., Author of "The Commonwealth of the Body."

No. 12.—**SYLLABUS OF LECTURES TO NURSES.**
By Andrew Davidson, M.D.

**COMPLETE CATALOGUE OF NURSING MANUALS POST FREE ON APPLICATION.**

**The Scientific Press, Ltd.,**

Publishers of Nursing Text-Books, Case-Books and Charts of all descriptions.

**28 & 29 SOUTHAMPTON STREET, STRAND, LONDON, W.C.**

